# Mammalian transposable elements and their impacts on genome evolution

**DOI:** 10.1007/s10577-017-9570-z

**Published:** 2018-02-01

**Authors:** Roy N. Platt, Michael W. Vandewege, David A. Ray

**Affiliations:** Department of Biological Sciences, Texas Tech University, Lubbock, TX USA

**Keywords:** Retrotransposons, Transposons, Mobile elements, TE defense, Horizontal transfer, Disease, Adaptation, Exaptation

## Abstract

Transposable elements (TEs) are genetic elements with the ability to mobilize and replicate themselves in a genome. Mammalian genomes are dominated by TEs, which can reach copy numbers in the hundreds of thousands. As a result, TEs have had significant impacts on mammalian evolution. Here we summarize the current understanding of TE content in mammal genomes and find that, with a few exceptions, most fall within a predictable range of observations. First, one third to one half of the genome is derived from TEs. Second, most mammalian genomes are dominated by LINE and SINE retrotransposons, more limited LTR retrotransposons, and minimal DNA transposon accumulation. Third, most mammal genome contains at least one family of actively accumulating retrotransposon. Finally, horizontal transfer of TEs among lineages is rare. TE exaptation events are being recognized with increasing frequency. Despite these beneficial aspects of TE content and activity, the majority of TE insertions are neutral or deleterious. To limit the deleterious effects of TE proliferation, the genome has evolved several defense mechanisms that act at the epigenetic, transcriptional, and post-transcriptional levels. The interaction between TEs and these defense mechanisms has led to an evolutionary arms race where TEs are suppressed, evolve to escape suppression, then are suppressed again as the defense mechanisms undergo compensatory change. The result is complex and constantly evolving interactions between TEs and host genomes.

## Introduction

Genome evolution is a highly dynamic process where large-scale genomic change can occur through a range of events including whole genome duplications, inversions, segmental duplications or deletions, and transposable element (TE) insertions and excisions (Cheng et al. 2005; Franke et al. 2017; Marques-Bonet et al. 2009; Ohno 1970). TEs are selfish genetic elements with the ability to mobilize within a genome. During the mobilization process, new copies of the TE can be created directly (Ostertag and Kazazian Jr. 2001), or indirectly, depending on the TE type or position of the TE relative to a DNA replication fork (Chen et al. 1992). TEs often reach high copy number over short evolutionary periods because of their replicative nature and continuous accumulation.

In most cases, TE insertions have no identified function (Biémont 2010) but examples of exapted TE insertions are becoming increasingly common (reviewed in Warren et al. 2015). Function has been ascribed to individual insertions (Mi et al. 2000), entire TE families (Bourque et al. 2008), and TEs in general (Cowley and Oakey 2013). Despite the potential advantages TEs provide, many insertions are neutral or deleterious, potentially resulting in a disease-state, or even a lethal allele. As a result, several genome defense mechanisms have evolved to limit TE activity. The goal of this review is to explore the role of TEs in mammal genome evolution. Below we discuss TE content, advantageous and deleterious effects of TE activity, and the evolution of TE defense strategies, all within a mammalian evolutionary context.

Our knowledge of mammalian genomics is relatively advanced compared to other vertebrates, yet we are still at a point where most of our results are derived from a handful of model taxa. Thus, broad conclusions may reflect clade-specific phenomena rather than generalizations to the entire class of mammals. However, as new sampling methods and sequencing technologies are developed, it will be possible to explore genomes increasing numbers of non-model mammals as well as TE dynamics at the population level to better understand the role of TEs in mammalian evolution.

## TE classification

**Fig. 1:**
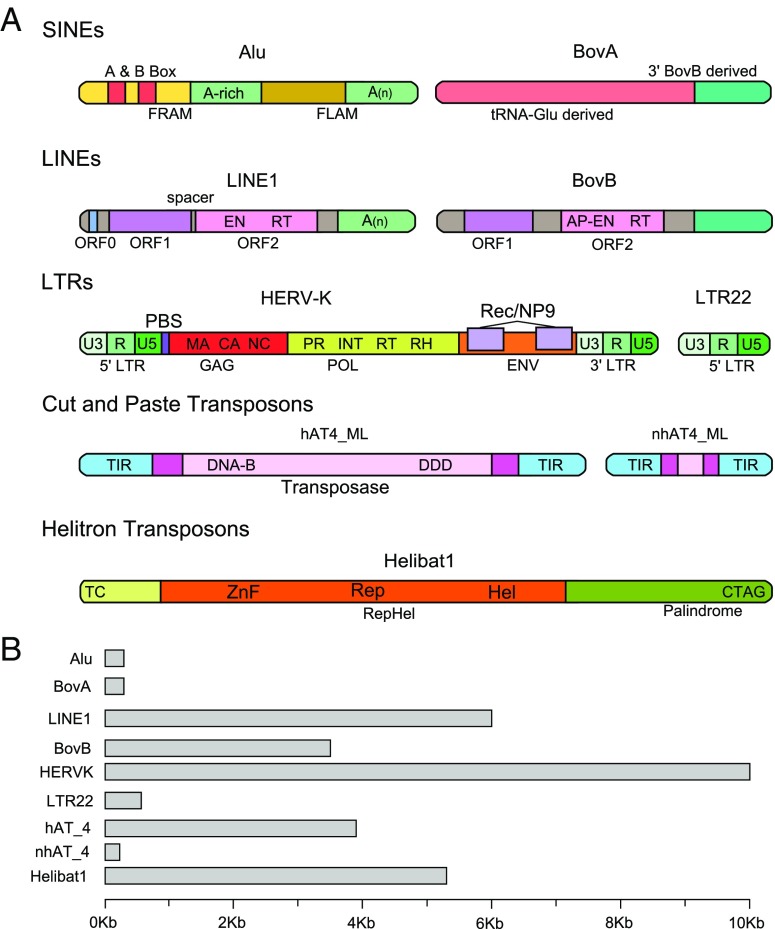
Mammalian transposable elements. **a** Structure of common mammalian transposable elements. A and B box, promoter regions derived from 7SL RNA; FRAM, free right Alu monomer; FLAM, free left Alu monomer; A(n) poly A repeat; UTR, untranslated region; ORF0, primate-specific open reading frame 0; ORF1, nuclear chaperone protein; ORF2 reverse transcriptase; EN, endonuclease domain; RT, reverse transcriptase domain; AP-EN, apurinic-apyrimidinic endonuclease; U3, unique 3′ sequence; R, repeated sequence; U5, unique 5′ sequence; PBS, tRNA primer binding site; GAG, GAG protein; MA, matrix domain; CA, capsid domain; NC, nucleocapsid domain; POL, polyprotein; PR, protease domain; INT, integrase domain; RH, RNAse H domain; ENV, envelope protein; Rec/NP9, Rec and NP9 proteins including possible alternative splicing events; TIR, terminal inverted repeat; DNA-B, DNA binding domain; DDD, three conserved aspartate residues; TC, TC dinucleotide sequence; ZnF, zinc-finger-containing motifs; RepHel, replicase protein; Rep, replicase domain; Hel, helicase domain; CTAG -CTAG nucleotide sequence. **b** Representative elements drawn to scale

TEs are generally classified into two groups based on their mobilization intermediates (Finnegan 1989). Class I elements, also known as retrotransposons, mobilize as an RNA intermediate. All retrotransposons, commonly called “copy and paste” elements, create new copies of themselves as they are reversely transcribed into the genome. Retrotransposons fall into two major groups, the long terminal repeat (LTR) elements and non-LTR elements, distinguished by the presence or absence of 100–300 bp direct terminal repeats (Fig. 1). The LTR elements, including endogenous retroviruses (ERVs), range in size from a few hundred base pairs to 10 Kb and are structured similarly to retroviruses (discussed below). Autonomous LTR elements encode at least a *gag* and *pol* protein, flanked by the long terminal repeats (LTRs) that give the elements their name. LTR retrotransposons mobilize and replicate through tRNA-primed template switching occurring within a viral-like particle encoded by the *gag* gene (Fig. 1; Leis et al. 1993; Levin 1995).

**Fig. 2:**
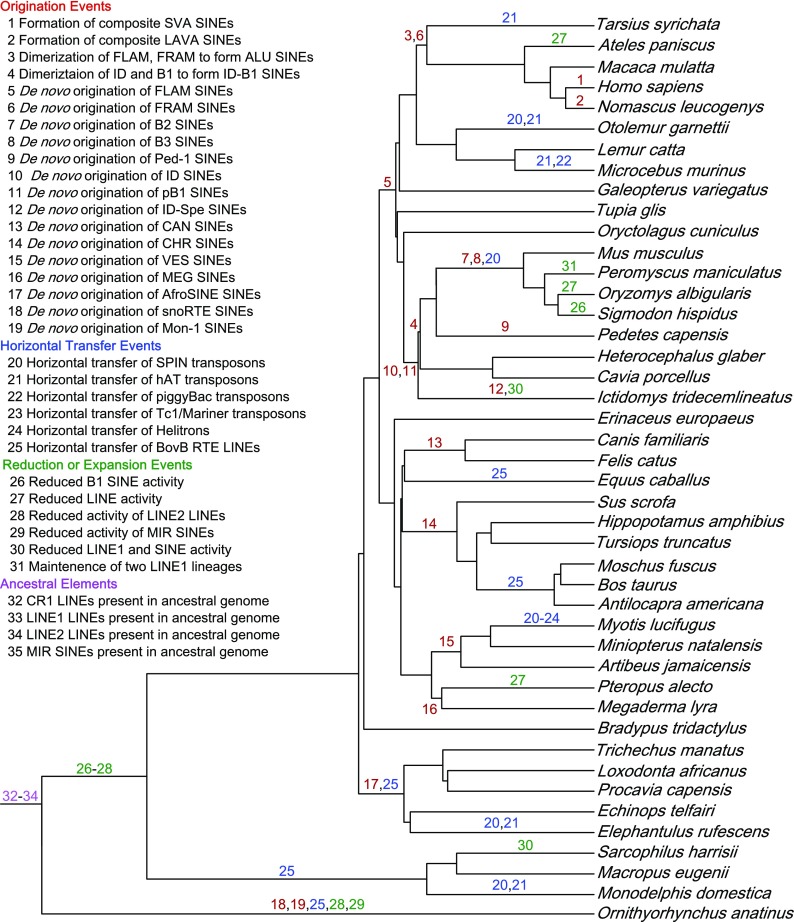
Major transitions in TE content along are plotted along the mammalian phylogeny. The mammal phylogeny is modified from Meredith et al. (Meredith et al. 2011). Events were inferred to specific nodes using information from (Alföldi et al. 2011; Churakov et al. 2010; Gogolevsky et al. 2008; Gogolevsky et al. 2009; Green et al. 2014; Hillier et al. 2004; Kriegs et al. 2007; Lupan et al. 2015; Nikaido et al. 2003; Novick et al. 2010; Pace et al. 2008; Pagan et al. 2010; Pritham and Feschotte 2007; Ray et al. 2015; Ray et al. 2006; Rinehart et al. 2005; Shimamura et al. 1999; Smit et al. 1995; Suh et al. 2014; Vassetzky and Kramerov 2002; Walsh et al. 2013; Wang et al. 2005; Warren et al. 2008) and are generally classified into four categories. “Origination Events” (red) refer to the de novo or composite origin of new TEs. “Horizontal Transfer Events” (blue) refer to the horizontal transmission of TEs from non-mammalian lineages. “Reduction or Expansion Events” (green) refer to dramatic shifts in accumulation patterns. “Ancestral Elements” (pink) refer to elements that were present in the ancestral mammalian genome

The non-LTR elements include long interspersed elements (LINEs) and short interspersed elements (SINEs). Both LINEs and SINEs can be identified by the presence of a repetitive tail, usually poly-A, and a lack of LTRs. LINEs are 4–7 Kb long and may encode between one and three proteins that provide the enzymatic machinery necessary for mobilization. The most common mammalian LINE, LINE1 (L1) contains two open reading frames (ORFs), a nuclear chaperone protein (ORF1) and a reverse transcriptase (ORF2; Fig. 1). A third very short protein (ORF0) was recently described in primate L1 elements but its function is unknown (Denli et al. 2015). SINEs can range in size from 150 to 500 bp and lack the machinery necessary for self-mobilization, i.e., non-autonomous (Fig. 1). Most mammal SINEs are derived from the combination of a 5′ head that is derived from a ribosomal or tRNA pseudogene and a 3′ tail homologous to a LINE. The LINE-like region of the SINE is used to parasitize the enzymatic machinery of LINEs for mobilization (Eickbush 1992). Unlike LINEs, de novo origination of SINEs is relatively common in mammals (Fig. 2).

Phylogenetic relationships estimated from the conserved residues of the RT domain indicate four distinct clades including LINE-like retrotransposons, Penelope-like retortransposons, prokaryotic retroelements (ex. group II introns) and the LTR containing retroelements, including LTR retrotransposons and retroviruses (Gladyshev and Arkhipova 2011). Retroviruses are structurally similar to LTR retrotransposons except for the addition of an env gene. The env gene codes an envelope protein which allows a retrovirus to potentially infect other cells. Env gene acquisition could be from recombination between infected hosts with active retrotransposition of LTR retrotransposons or the acquisition of modification of a host encoded gene (Eickbush and Malik 2002; Koonin et al. 1991). These events have likely occurred multiple times leading to the independent origins of retroviruses with some groups obtaining the ability to leave the cell and infect others including the caulimoviruses and gypsy viruses (Eickbush and Jamburuthugoda 2008; Herédia et al. 2004). Once a germ cell is infected, the retrovirus becomes endogenized and can then be transmitted vertically from parent to offspring.

Class II elements, also known as the DNA transposons, mobilize as a DNA intermediate associated with a transposase. DNA transposons can be subdivided into two major groups; the cut-and-paste and rolling-circle transposons (Wicker et al. 2007). Cut-and-paste elements excise themselves from the genome as a double-stranded DNA intermediate associated with a transposase, an enzyme encoded by autonomous instances of the element family in question. Common cut-and-paste transposons found in mammals include the Tc1/*mariner*, *h*ATs, and *piggyBac* families, all of which can be characterized by their terminal inverted repeats–ranging in size from 10 to1,000 bp–and the catalytic domains of their transposase (Fig. 1; Feschotte and Pritham 2007). Rolling-circle transposons, or *Helitrons*, mobilize as a single-stranded DNA copying itself via rolling-circle replication (Kapitonov and Jurka 2001). Autonomous *Helitrons* contain a RepHel protein and a ~ 20 bp palindrome that functions as a termination sequence 10–20 bp from the 3′ end of the element (Fig. 1; Kapitonov and Jurka 2007).

## Mammalian TE content and evolution

A survey of TEs from species spanning the mammalian phylogeny (Meredith et al. 2011) can be used to generalize TE content in mammals as a whole (Fig. 2). Under this assumption, we can make the following observations regarding TE content and dynamics in mammalian genomes:

- One half to one third of the mammal genome is derived from TEs.
- LINE and SINE retrotransposons are the most common types of TE. DNA transposons are rare and/or ancient.
- Mammals usually have one or more actively mobilizing TE family
- Horizontal transfer of TEs is rare in mammals

As with any observations made across a group as diverse as mammals and a phenomenon as dynamic as TEs, there are exceptions to each of these observations. Data supporting the above observations plus the exceptions are described below.

### One half to one third of the genome is derived from TEs

**Fig. 3:**
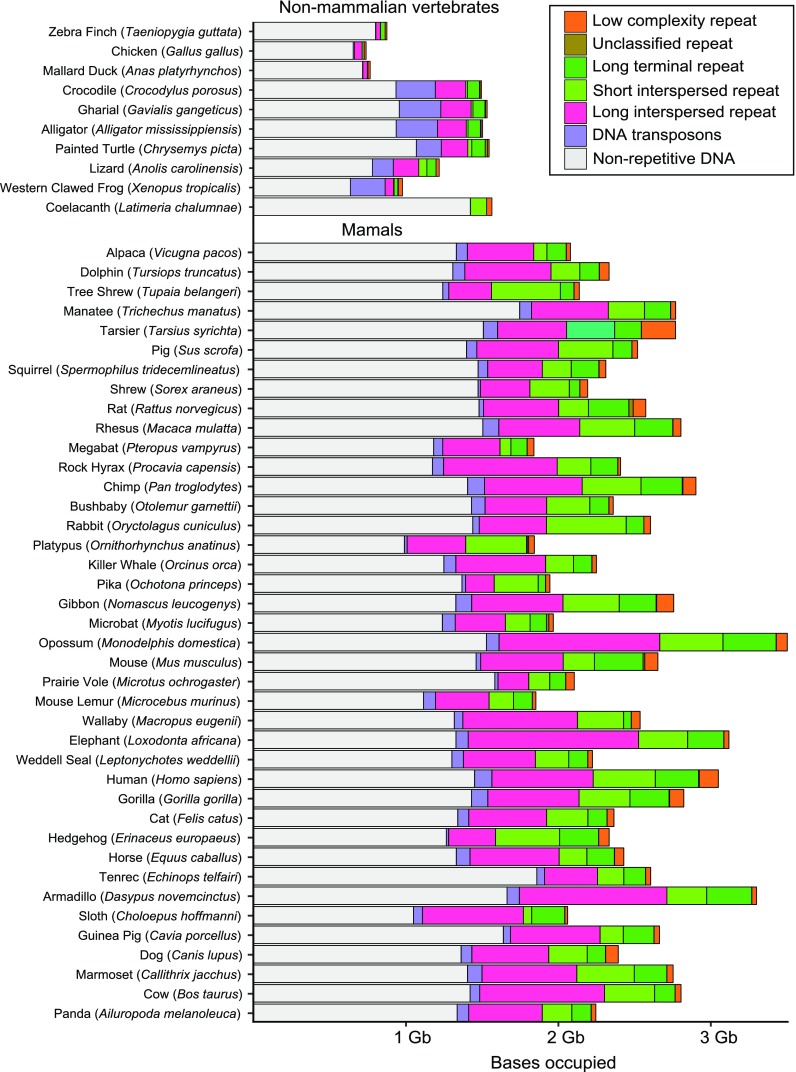
TE content in mammal and non-mammal vertebrate genomes. TE content was quantified from pre-masked genomes available at http://repeatmasker.org/genomicDatasets/RMGenomicDatasets.html (last accessed 30 November 2014). Genome size was estimated from the number of bases in the genome assembly

TEs typically make up between one third and one half of mammal genomes (Fig. 3; Elsik et al. 2009; Lander et al. 2001; Mikkelsen et al. 2007; Miller et al. 2008; Warren et al. 2008; Waterston and Pachter 2002) but it is likely that estimates of TE content are biased downwards based on computational and methodological limitations of TE identification. At the time of insertion, novel TE insertions are identical, or nearly identical to, the parent insertion. As neutrally evolving TE insertions age, the genetic distance between insertions increases. Ancient insertions with less than 50% sequence similarity to a query TE rapidly become unidentifiable using homology-based methods. In addition to natural sequence divergence between TE insertions, homology-based searches may not identify lineage-specific repeats if they are not defined a priori (Platt II et al. 2016a). De novo TE identifications tend to be more accurate and can be used to identify particularly ancient elements. For comparison, current estimates of TE content in the human genome range from 49% to as high as 69% when using homology or de novo-based searches, respectively (de Koning et al. 2011). Because of sequence degradation and homology-based limitations, estimates of TE content are always biased against older elements and sometimes against newer elements. It is almost certain that mammalian genomes are more repeat rich than currently recognized.

### LINEs and SINEs are more abundant than LTR elements and DNA transposons

The repetitive portion of mammal genomes is dominated by LINEs and SINEs, followed by LTR retrotransposons, and then DNA transposons. In most, ~ 75% of the repetitive portion is derived from non-LTR retrotransposons (Fig. 3; Lander et al. 2001; Waterston and Pachter 2002). Non-LTR retrotransposons in the platypus genome make up 97.4% of all repetitive sequences and LINEs by themselves occupy 20% of the genome (Warren et al. 2008). The LINE-1 (L1) family is the most successful TE family in mammals, and frequently occupies hundreds of megabases in therian genomes. SINE expansions in mammals piggyback on the success of their autonomous LINE partners. Rather than continuous expansion from a single SINE family, as in the case of L1, unique and lineage-specific SINE families have arisen multiple times (Fig. 2; Kramerov and Vassetzky 2011).

LTR retrotransposons are present in mammal genomes at moderately high copy number, and can occupy between ~4 and 10% of the genome (Mikkelsen et al. 2007). However, the accumulation of LTR elements in the genome may not reflect past activity because recombination between the terminal repeats of LTR elements can remove nearly the entire element, leaving behind a solitary LTR (Bennetzen and Kellogg 1997; Smit 1993).

DNA transposons are usually present in low copy numbers relative to retrotransposons, occupying less than 3% of mammalian genomes (Platt II and Ray 2012). Low copy number of DNA transposons is driven in part by two factors. First, most mammals lack autonomous class II elements (Pace and Feschotte 2007), so the DNA transposons that are present in mammalian genomes are decaying vestiges of earlier transposition events. Second, if a cut-and-paste DNA transposon is active, a new copy is only created if it reinserts in front of a replication fork. To date, the vespertilionid bats are the only mammals with significant, active DNA transposition (discussed below).

### Most mammals have one or more actively mobilizing TE family

The vast majority of TE insertions in mammal genomes are incapable of mobilization (Fanning 1983). With L1 elements, this is true primarily because most insertions are truncated at the 5′ end due to inefficient reverse transcription (Grimaldi et al. 1984). Furthermore, de novo insertions of any type may be mutated during the insertion process or targeted for transcriptional and post-transcriptional silencing by one of several defense mechanisms (described below). So, while there may be tens of thousands of copies of any TE present, only a small fraction is capable of mobilizing at any moment. In humans and mice, only 6 of more than ~868,000 and 2382 of ~599,000 L1s are retrocompetent in their respective genomes (Brouha et al. 2003; Zemojtel et al. 2007).

Despite the limited number of retrocompetent elements, retrotransposition of LINEs and/or SINEs persists in mouse, human, and most other mammal genomes examined to date. TE quiescence of LINEs and/or SINES has only been observed in the ground squirrel (Platt II and Ray 2012), Tasmanian devil (Nilsson 2016), *Ateles* spider monkeys (Boissinot et al. 2004), sigmodontine rodents (Grahn et al. 2005; Rinehart et al. 2005), and pteropodid bats (Cantrell et al. 2008) but, given the timing and phylogenetic distribution of these silencing events, it is plausible that quiescence has impacted in as many as 15% of all mammal species (Platt II and Ray 2012). Understanding the genomic mechanisms, population genetic parameters, and random factors that reduce or eliminate TE activity is one of the major questions in vertebrate genome evolution (Goodier 2016).

### Horizontal transfer of TEs is relatively rare

TEs in mammals are spread primarily through vertical inheritance, though the mobile nature of TEs means the insertion patterns may vary among lineages. In some rare instances, TEs are horizontally transferred among taxa. In mammals, there are fewer than 20 documented horizontal transfer (HT) events in the last 160 MY, compared to 2248 in the last 10 MY in insects (Fig. 2; Peccoud et al. 2017). The most successful HT event in mammals was the expansion of BovB LINES into a diverse group of mammals including afrotherians (ex. elephants and tenerecs), ruminants (ex. cattle and deer), marsupials (ex. kangaroos and possums), and protherians (platypuses and echidnas). The expansion of BovB in mammals is the result of at least four separate transfer events (Walsh et al. 2013) as recently as 50 MYA (Kordis and Gubensek 1998) likely from a parasite vector (Walsh et al. 2013). Despite the relatively recent invasion into an ancestral ruminant, BovB LINEs and SINEs make up as much as 18.4% (Elsik et al. 2009) and 10.37% (Ge et al. 2013) of the bovid genome, respectively.

Various DNA transposon families have successfully invaded mammalian genomes but not accumulated as successfully as the BovBs. HT of SPIN transposons occurred between 15 and 46 MYA into the galago, murine rodents, opossum, tenerec, and vespertilionid bats (Pace et al. 2008), *hAT*s were transferred to the opossum, tenerec, some primates, and vespertilionid bats (Novick et al. 2010) and *piggyBac*s were transferred into the mouse lemur and vesperitionlind bats (Pagan et al. 2010). The frequency of these events and the fact that many occur in the same taxa suggest that some species may be more susceptible to HT than others, most notably the vespertilionid bats, who also have experienced HT of *Helitrons*, and Tc1/*mariners*. The HT of *hAT*s, *piggyBac*s, Tc1/*mariner*s, and *Helitron* transposons into these bats occurred over a series of events within a narrow 10–20 MY window (Platt II et al. 2016b; Pritham and Feschotte 2007; Ray et al. 2008; Ray et al. 2006; Thomas et al. 2011). As a result, almost half of all recent transposition in the vesper bats has been from DNA transposons compared to less than 1% in other laurasiatherians (Platt II et al. 2014). Still, DNA transposons only account for 3–5% of vespertilionid genomes despite the recent increase in accumulation (Pagán et al. 2012).

## Impacts of advantageous TE insertions on mammalian genomes

The presence of TEs and their dynamic nature has shaped mammal genomes in significant ways (reviewed in Chalopin et al. 2015; Sotero-Caio et al. 2017; Warren et al. 2015). Below we describe some advantageous and deleterious effects of TE activity and content. We emphasize that despite our focus on the selectively advantageous or deleterious impacts of TEs, many TE insertions have accumulated through non-adaptive processes associated with reduced effective population sizes and the resulting increased effects of genetic drift (Lynch and Conery 2003).

### Exaptation of TEs

More than a quarter million conserved non-coding elements are derived from TEs in the human genome alone (Lowe and Haussler 2012). Further, identification of additional exaptation events, notably TEs as regulatory units, is becoming increasingly common. Some TE’s promoters contain transcription factor binding sites and other regulatory motifs (Bourque et al. 2008). As TEs mobilize in the genome, they spread their own regulatory motifs to new loci. If a TE inserts near-promoter regions of other genes, selection can then co-opt the TE’s regulatory elements to alter gene expression of the nearby genes. Given enough time, a novel regulatory network can emerge where a single transcription factor, originally associated with TE transcription, may link dozens, or even hundreds, of previously unrelated genes (Chuong et al. 2017). As selection acts on the nascent network, it can become highly specialized. In the case of MER20, binding sites for hormone responsiveness and pregnancy-related transcription factors found within the TE itself were spread throughout the genome of the ancestral placental mammal.

Multiple examples exist including the differentiation of endometrial cells in the presence of progesterone, which was a critical step in the evolution of pregnancy in placental mammals. Thirteen percent of genes associated with differentiation of endometrial stromal cells appear to be regulated by motifs found in a eutherian-specific *hAT* transposon, MER20. In addition, almost half of all MER20s in the human genome are found within 200 Kb of progesterone responsive genes (Lynch et al. 2011). In some instances, MER20 would insert next genes providing transcription factor-binding sites (or epigenetic modifications) that may not have previously affected that genes. As this continued to happen, a complex network of genes, partially regulated by MER20-derived regulatory sites, developed into a cell-type specific regulatory network for differentiation of endometrial cells (Lynch et al. 2011; Lynch et al. 2015). In another example, three TEs, an AmnSINE, X6b_DNA transposon, and MER117 hAT inserted adjacent to each other in a sequential manner to form a complex promoter for secondary palate development in eutherian mammals. None of the insertions exhibit promoter activity on their own but instead work cooperatively to regulate *wnt5* expression (Nishihara et al. 2016). Chuong et al. (2017) provides a more detailed review of TE-driven regulatory networks.

Co-option of LTRs is inversely proportional to the age of the element subfamily; younger elements are more likely to be co-opted, a trend that contrasts with the co-option rate of other TEs (Franke et al. 2017). LTRs can serve as gene-remodeling platforms where the promoters and initial exons of a gene are derived from an LTR. LTR-derived promoters and 5′ exons are incorporated into 842 protein coding and lncRNA genes expressed during the transition from oocyte to zygote in rodents (Franke et al. 2017). In mice an MT-C LTR insertion into a DICER intron has truncated the first 6 exons, provided an alternative promoter and novel first exon. This DICER isoform has acquired oocyte-specific expression and is essential for fertility (Flemr et al. 2013).

### TEs may promote adaptability

TEs can alter gene expression, disrupt coding genes, transduce exons, or promote recombination allowing for dramatic and rapid restructuring of the genome that may exceed the changes offered by point mutations. These changes may allow populations to more fully explore a fitness landscape in a shorter period of time; increasing the “adaptability” of the population (Casacuberta and González 2013). The role of TEs in promoting adaptability has been explored theoretically (Werren 2011), in the laboratory (Stoebel and Dorman 2010), observed in the wild (Schrader et al. 2014), and has become a critical framework to understand invasion genetics (Stapley et al. 2015), but has yet to be demonstrated in a mammalia. Two hypotheses regarding the role of TEs in promoting adaptability are directly associated with mammals; the stress-response (McClintock 1984) and TE Thrust hypotheses (Oliver and Greene 2011). A variant of the stress-response hypothesis, the epi-transposon hypothesis (Zeh et al. 2009), posits that during times of environmental stress epigenetic suppression of TEs is relaxed allowing for burst of TE activity. The increased rates of TE activity allow populations to explore the fitness landscape. The epi-transposon hypothesis has been explored more completely in plant studies (for examples see Ito et al. 2016; Nozawa et al. 2017) than in vertebrates. Despite these limitations general observations in the human genome, including increases in TE expression due to chemical exposure, approximating environmental stress (Kale et al. 2005), and the association of TEs in stress related gene regions (van de Lagemaat et al. 2003), tend to support tenets of the epi-transposons hypothesis. The TE-Thrust hypothesis proposes that lineages with TE activity are more fecund than those without, and is based on the observation that TE accumulation tends to be associated with novel genetic change. Like the epi-transposon hypothesis, it is difficult to directly test the TE-Thrust hypothesis. Instead, correlations between TE activity with major evolutionary innovations in mammalian lineages represent the best supporting evidence (Brandt et al. 2005; Lowe and Haussler 2012; Mikkelsen et al. 2007; Pace and Feschotte 2007; Platt II et al. 2014; Suh et al. 2014). However, given that TE activity is more likely to lead to declines in fitness reductions rather than increases in fitness, a rigorous test of the assumption that TE activity leads to as an example, increased diversification rates, to be tested within a phylogenetic context.

## Impacts of deleterious TEs insertions on mammalian genomes

Around 10% of all de novo mutations in lab mice are the result of TE insertions (Maksakova et al. 2006). In fact, the mutagenic power of TEs is so great that transposons are often used to identify gene function in model organisms including humans and mice (Dupuy et al. 2005). The deleterious effects of TE activity can result in reduced fitness in populations. Below we discuss the evidence of and known deleterious effects of TEs in mammals, most of which is derived from studies in human and mouse models.

### Selection drives TE distribution

One difficulty in identifying deleterious insertions is that they are often lethal or only slightly deleterious and present no obvious phenotypes. As a result, our knowledge of deleterious TE insertions is more limited than one might expect given the frequency with which deleterious TE insertions are expected to arise. Rather than directly observing the deleterious effects at the morphological level, the deleterious nature of TE insertions can be inferred from biases in their distribution across the genome.

Surveys of the human genome show that TE insertions are not randomly distributed. Younger retrotransposon insertions are biased toward AT rich regions reflecting their target-site preference (Medstrand et al. 2002). As Alu elements age, their density in GC rich regions increases relative to L1 (Jurka et al. 2004). In vertebrates, GC rich regions are typically associated with high gene density compared to the relatively gene poor AT rich regions. Accumulation of elements in GC rich regions likely reflects selection against inter-TE recombination, the only mechanism thought to remove TEs, since such events are more likely to disrupt coding regions than to salvage them (Abrusán and Krambeck 2006; for an alternative explanation see Kvikstad and Makova 2010; Medstrand et al. 2002). If young insertions are neutral or slightly deleterious, selection should remove the slightly deleterious insertions. Under this assumption, older elements are more likely to be neutral than their younger counterparts. Because of GC-biased accumulation, TEs are found in introns of almost 90% of human and mouse genes. Intronic TE insertions tend to be located more than 150 bp away from the closest exons and in the opposite orientation of the gene (Burns and Boeke 2012). These positional biases reflect selection against insertions that disrupt splice sites and/or generate anti-sense exonic transcripts via read-through transcription from promoters in the TE insertion. Interestingly, most known mutagenic insertions found in introns violate these patterns (Zhang et al. 2011).

### TE contribution to mutational meltdown

While some levels of TE activity may promote adaptation (discussed above), it is possible that excess TE activity can contribute to mutational meltdown of populations. Mutational meltdown is a positive feedback loop where deleterious mutations accumulate in populations leading to decreased fitness and reduced population sizes which are more prone to the accumulation of additional deleterious mutations via drift. Mutational meltdown is difficult to observe directly because it is a feed-forward loop that results in extinction. As a result, the literature on mutational meltdown is biased toward theoretical work (Lynch et al. 1995) or experiments with lab populations (Zeyl et al. 2001), and less is known about the process in wild species with long generation times (Rowe and Beebee 2003). The best example of mutational meltdown in mammals is from the Wrangel Island mammoth genome. The single genome available for this group shows an accumulation of gene deletions, premature stop codons, and reduced heterozygosity compared to other mammoth genomes from individuals in larger populations (Rogers and Slatkin 2017). In addition, the Wrangel Island mammoth has an excess of retrogenes. Because retrogenes are a direct byproduct of LINE activity, their presence indicates a burst of retrotransposon activity just prior to extinction of the Wrangle Island mammoth population (Rogers and Slatkin 2017). This final burst of TE activity, beyond creating retrogenes, could have contributed to the mutational meltdown of the Wrangel Island mammoth population. Interestingly, this burst of TE activity is expected under the epi-transposon hypothesis (Zeh et al. 2009).

### Somatic diseases

TE expression was previously thought to be limited to the germ line but recent studies identified extensive TE expression in somatic tissue. When occurring in the germ line, lethal insertions are purged, but somatic stem cell insertions are more tolerable and associated with cancer, neuropathy, and the aging process.

TE insertions have been associated with more than 100 diseases (Chénais 2013; Hancks and Kazazian 2016) including several forms of cancer. TE-induced cancer can arise by altering tumor suppressor genes or proto-oncogenes (Morse et al. 1988). In each case, disruption of one allele by a TE usually needs to coincide with a loss-of-function mutation at the other allele before tumorigenesis, so the prevalence of TE-induced tumorigenesis is unknown (Burns and Boeke 2012). Still, examples of TE-driven tumorigenesis exist. For example, an L1 element insertion into the APC tumor suppressor gene initiates colorectal cancer (Scott et al. 2016). Splice variants of Rec and Np9 from the *env* gene in HERV-Ks bind and suppress the promyelocytic leukemia zinc finger protein which is a transcriptional repressor of the oncogene c-myc (Denne et al. 2007). Changes in genome structure including chromosomal translocations, recombination between, and duplications driven by Alu insertions have all been associated with several forms of leukemia (Jeffs et al. 1998; O'Neil et al. 2007; Strout et al. 1998).

Neural tissues contain unexpected levels of TE expression. More than 2200 somatic TE insertions were identified in just three individuals, a large number of which tended to be within or in close proximity to protein-coding genes (Baillie et al. 2011). It has been estimated that 1 in every 300 neuronal genomes contains a novel L1 insertion (Evrony et al. 2016), which means that the typical mammal brain may contain millions, possibly billions of novel TE insertions. These and other observations seem to imply that some level of TE expression is necessary for normal neuronal development, yet progenitor cells are vulnerable to accumulating deleterious mutations (Li et al. 2012; Reilly et al. 2013). Since TEs are going to insert into open, euchromatin sites, they are likely to insert near, or potentially into, transcribed genes associated with neural function (reviewed in Nekrutenko and Li 2001). Environmental stimuli ranging from exposure to light (deHaro et al. 2014), heavy metals (Kale et al. 2005), aromatic hydrocarbons (Stribinskis and Ramos 2006), even physical exercise (Muotri et al. 2009), can potentially increase L1 expression beyond the already elevated levels in neural tissues. As a result, neural tissues show increased accumulation of TE insertions in genes associated with stress, including alcoholism and post-traumatic stress disorder (Reilly et al. 2013). In humans, Alu elements retrotransposed into the mitochondrial, TOMM40 gene 16 times leading to a serious of conformational changes and/or truncated proteins that are less than fully functional (Larsen et al. 2017). Mitochondrial dysfunction in neural cells likely leads to increased oxidative stress and subsequent inflammatory response imitating a feedforward loop that leads to reduced neural function and disease (Swerdlow and Khan 2004).

## Mammalian protection against TEs

Because TEs are capable of compromising genome integrity, disrupting gene function, and inducing disease states the genome has evolved several, semi-redundant defensive systems to limit TE activity. These systems range from transcriptional silencing to transcript editing. Below we present three defense mechanisms in mammalian genomes and discuss how they have shaped mammalian genome evolution.

### KRAB/KAP1 histone modification

Retrotransposons are transcriptionally silenced in early embryos by histone modification and DNA methylation, although the initiators of this process have, until recently, been unknown. Krüpell-associated box (KRAB) domain-containing zinc finger proteins (KRAB-ZFPs) have sequence-specific DNA binding ability via C-terminal zinc fingers (Urrutia 2003) that are used to recognize retrotransposons in early embryos (Rowe et al. 2010). After recognition, KRAB-ZFPs recruit KRAB-associated protein 1 (KAP1), which in turn can bind to any one of a series of epigenetic regulators, including, histone methyltransferases (ESET), heterochromatin protein 1 (HP1), nucleosome remodeling and deactylation (NuRD) complex, and DNMT3A and DNMT3B which methylates DNA (Ecco et al. 2017; Feschotte and Gilbert 2012). Knocking out KAP1 in early mouse embryos leads to an upregulation of ERV retrotransposons (Rowe et al. 2010).

### Sequence editing with APOBECs

The APOBEC family of cytidine deaminases act by editing reverse (cDNA) transcripts (Friedli and Trono 2015; Mangeat et al. 2003). The enzymes mediate the deamination of cytosine to uracil, causing either direct destruction or debilitating levels of hypermutation in the TE cDNA (Harris et al. 2003). APOBECs arose in early vertebrates, but at least two subfamilies of APOBECs (APOBEC3 and APOBEC1) arose through gene duplications in early mammals, and APOBEC3 is only present in placental mammals (Conticello 2008; Conticello et al. 2004; Rogozin et al. 2007). The family member APOBEC3G was first noticed to edit viral cDNA from HIV lacking the *vif* gene and murine leukemia virus (Bishop et al. 2004). ERVs are structurally like these retroviruses and are also in fact edited by APOBECs. It was first hypothesized that non-LTR retroelements would not be affected by APOBECs because APOBEC3G is restricted to the cytoplasm and non-LTR reverse transcription occurs in the nucleus. However, it was later discovered that several APOBEC3s are expressed in the nucleus and the family inhibits a broad range of both LTR and non-LTR retroelements (Bogerd et al. 2006; Friedli and Trono 2015; Kinomoto et al. 2007; Richardson et al. 2014; Schumann 2007).

### PIWI proteins and piRNAs

The typical mammalian genome encodes four PIWI proteins: PIWIL1 (MIWI), PIWIL2 (MILI), PIWIL3, and PIWIL4 (MIWI2). Their partners, piRNAs, are the most abundant small RNA in testis and range from approximately 24 to 32 bases. piRNAs have few distinguishing characteristics except for a uridine bias in the first position of sense-oriented sequences. PIWI proteins and the associated piRNAs are predominately expressed in the germ line and are required for spermatogenesis (Aravin et al. 2006; Carmell et al. 2007; Kuramochi-Miyagawa et al. 2004; Lau et al. 2006).

PIWIs silence TEs through two pathways, direct cleavage of TE transcripts and de novo methylation of TE loci, both of which are dependent on the “ping-pong” cycle where PIWIs use piRNAs as guides to TE transcripts. Briefly, sense primary piRNAs direct PIWIL2 to complementary anti-sense TE-derived transcripts. These transcripts are cleaved to generate anti-sense secondary piRNAs and these secondary piRNA are incorporated into a PIWIL4 or PIWIL2 complex which is guided to a sense TE transcript, and the cycle is repeated creating a feed forward loop that increases the number of piRNA guides and reduces the abundance of TE mRNAs. During testis development, genome-wide methylation marks are erased and reset in primordial germ cells. As a result, TEs are released from epigenetic silencing and their expression increases (Molaro et al. 2014). During this time, PIWIL4 is thought to mark TE loci for downstream de novo methylation by methyltransferases DNMT3L and DNMT3A (Aravin et al. 2008; Molaro et al. 2014); however, this exact mechanism is unknown. Knocking out PIWIL4 and PIWIL2 lead to upregulation of retrotransposon in the male germ line, an arrest of gametogenesis, and complete sterility in male mice, likely due to unrestricted retrotransposon mobilization (Aravin et al. 2007; Carmell et al. 2007).

As a primary defense mechanism against TE proliferation, PIWI processing likely affects TE composition in mammalian genomes (Vandewege et al. 2016). When comparing TE expression and piRNA processing in mammals with different active TE families, a strong positive relationship between piRNA and TE transcript abundance was present; more piRNAs were derived from highly expressed TEs. The relationships between TE transcription and piRNA quantity, however, did not correlate with “efficiency” in targeting. Young SINE expression in the dog and horse genomes is comparable, yet young SINEs accumulate at a much greater rate in the dog than the horse genome. SINE transcripts in the horse are targeted, cleaved, and/or methylated allowing for high SINE expression but limited accumulation when compared to dogs. This reduced efficiency of PIWI processing in the dog genome has resulted in 166,148 SINEC_Cfa SINE insertions versus 53,092 ERE1 in the horse over a relatively similar amount of time and despite similar SINE transcription levels (Vandewege et al. 2016). SINE accumulation is so rapid in the dog that ~ 10,000 bimorphic loci in the domestic dog population (Wang and Kirkness 2005).

### The genomic arms race with TEs

TEs and genomic defenses systems are engaged in an arms race that mirrors the relationship between pathogens and the immune system. The genome must constantly develop strategies to fight transposition, which pressures TEs to evolve to escape repression. For example, by resembling regulatory sequences, some LTRs likely escape methylation in embryonic stem cells (Gerdes et al. 2016). With the exception of birds, there are hundreds of KRAB-ZFPs encoded by most vertebrate genomes (Emerson and Thomas 2009; Liu et al. 2014). KRAB-ZFPs experience rapid evolutionary changes in zinc finger structure, sequence, and expression, and splicing patterns (Nowick et al. 2010) and tandem duplication events drive the rapid expansion of the KRAB-ZFP gene families. Further, there is a positive correlation between the number and age KRAB-ZFPs and genomes ERV content (Thomas and Schneider 2011). Selection is also a strong driver of evolution in APOBEC sequences (Sawyer et al. 2004). APOBEC3G has been under strong positive selection in primates, and additional members of the APOBEC family display strong signals of positive selection in humans (Sawyer et al. 2004). Primate genomes encode the most APOBECs, and the expansion of this family during primate evolution coincides with a decrease in TE activity (Schumann 2007). The PIWIs are fundamentally different from APOBECs and KRAB-ZFPs, where selection is a strong driver of their evolution. The sequences and structure of PIWI proteins are well conserved, but the targeting mechanism, the piRNAs are directly processed from active TEs allowing the inhering targeting and silencing of the newest and most expressed TEs (Molaro et al. 2014; Vandewege et al. 2016).

The genomic TE defense system is overall adaptive and redundant given there are defenses at every stage of the TE replication cycle. TEs are silenced via KRAB-ZFPs during embryogenesis and PIWI proteins methylate and silence young TEs during testis development. Elements that escape methylation and become expressed are cleaved in the cytoplasm by additional PIWI proteins. And if a TE mRNA is not cleaved, APOBECs edit the transcript so that the new insertion is no longer functional. These pathways, and others just being described (Martinez et al. 2017; Schorn et al. 2017), work together to prevent full-length autonomous elements from propagating in mammalian genomes.

## Conclusion

One third to one half of the typical mammalian genome is derived from TEs, primarily non-LTR retrotransposons. Because of their abundance, TEs can have significant impacts on mammalian genome evolution. Active (retro)transposition can provide opportunities for expatiation events, build novel regulatory networks, and even increase the adaptive potential of a population. Despite these benefits, many insertions with any phenotypic impact are neutral or deleterious. Highly deleterious insertions will be rapidly purged from the gene pool; however, somatic insertions can arise presenting as any number of cancers or neurological diseases. To mitigate the potential deleterious effects of TE activity, several redundant defense mechanisms have evolved to limit TE expression.

Our knowledge of mammalian genomics is relatively advanced compared to other vertebrates, yet we are still at a point where most of our results are derived from a handful of model taxa. At this point, broad conclusions may reflect clade-specific phenomena rather than generalize to the entire class of mammals. As new sampling methods and sequencing technologies are developed, it will become possible to explore genomes of non-model, mammalian tax at the population level to truly understand the role of TEs in mammalian evolution.

## Abbreviations

TE: Transposable element
ERV: Endogenous retrovirus
LTR: Long terminal repeat
LINE: Long interspersed element
SINE: Short interspersed element
Kb: Kilobases
Mb: Megabases
Bp: Base pairs
ORF: Open reading frame
L1: LINE1
L2: LINE2
RTE: RNA transport element
MY: Million years
MYA: Million years ago
piRNA: PIWI-interacting RNA
KRAB-ZFPs: KRAB zinc finger proteins
APOBEC: Apolipoprotein B mRNA editing enzyme catalytic polypeptide-like
